# IL35 modulation altered survival, cytokine environment and histopathological consequences during malaria infection in mice

**DOI:** 10.1186/s12936-019-3070-x

**Published:** 2019-12-19

**Authors:** Ramatu Omenesa Bello, Maizaton Atmadini Abdullah, Roslaini Abd Majid, Voon Kin Chin, Mohammed Faruq Abd Rachman Isnadi, Zaid Osama Ibraheem, Mohd Khairi Hussain, Mohammed Garba Magaji, Rusliza Basir

**Affiliations:** 1 Department of Human Anatomy, Faculty of Medicine and Health Sciences, University Putra Malaysia, 43400 Serdang, Selangor Malaysia; 2 Department of Pathology, Faculty of Medicine and Health Sciences, University Putra Malaysia, 43400 Serdang, Selangor Malaysia; 3 Department of Medical Microbiology and Parasitology, Faculty of Medicine and Health Sciences, University Putra Malaysia, 43400 Serdang, Selangor Malaysia; 4 School of Biosciences, Faculty of Health and Medical Sciences, Taylor’s University Lakeside Campus, 47500 Subang Jaya, Malaysia; 5 Department of Pharmacology, Faculty of Pharmacy, Al Rafidain University, Al Mustansyria, Baghdad, Iraq; 6 Department of Biomedical Sciences, Faculty of Medicine and Health Sciences, University Putra Malaysia, 43400 Serdang, Selangor Malaysia; 7 Department of Pharmacology and Therapeutics, Faculty of Pharmaceutical Sciences, Ahmadu Bello University, Kaduna, 810107 Nigeria

**Keywords:** *Plasmodium berghei*, Interleukin-35, Immunohistochemistry, Cytokines

## Abstract

**Background:**

The immune modulating potential of IL-35 in multiple human disorders has been reported. Consequent upon the recognition of inflammatory cytokine activation and its preponderance for mediating pathology during malaria infection, the study aimed to characterize the expression and functional contribution(s) of IL-35 in *Plasmodium berghei* (strain ANKA) infected mice.

**Methods:**

*Plasmodium berghei* infection in male ICR mice was used as the rodent model of choice. The time course of IL-35 expression in the systemic circulation and tissues of *P. berghei* infected mice as well as their healthy control counterparts was assessed by enzyme linked immunosorbent assay and immunohistochemistry respectively. The effect of modulating IL-35 by recombinant IL-35 protein or neutralizing anti-Epstein-Barr virus-induced gene 3 antibody on the cytokine environment during *P. berghei* infection was assessed by flow cytometry. Furthermore, the influence of modulating IL-35 on histopathological hallmarks of malaria and disease progression was evaluated.

**Results:**

Interleukin-35 was significantly up regulated in serum and tissues of *P. berghei* infected mice and correlated with parasitaemia. Neutralization of IL-35 significantly enhanced the release of IFN-γ, decreased the expression of IL-6 and decreased parasitaemia patency. Neutralization of IL-35 was also associated with a tendency towards increased survival as well as the absence of pathological features associated with malaria infection unlike recombinant IL-35 protein administration which sustained a normal course of infection and unfavourable malaria associated histological outcomes in *P. berghei* infected mice.

**Conclusion:**

These results indicate the involvement of IL-35 in *P. berghei* induced malaria infection. IL-35 neutralization strategies may represent viable therapeutic modalities beneficial for the resolution of malaria infection.

## Background

Numerous responses during malaria infection are influenced by interaction(s) between the immune system of the host and the *Plasmodium* sp. [1]. Severe malaria infection caused by *Plasmodium falciparum* typically features overt systemic derangement associated with vital organ dysfunction and possibly death [2–4]. The majority of severe malaria infections accrue poor prognosis and have been evinced to result from persistent hyper activation of the immune system by cytokines triggered in response to parasite invasion (and parasite replication) viz parasite-driven responses [5]. Cerebral malaria represents the most drastic clinical consequence of severe malaria among a succession of overlapping clinical syndromes including severe anaemia, respiratory distress and acute kidney failure [6].

The mechanisms underlying exaggerated harmful cytokine activation during malaria have been attributed to excessive stimulation of the immune system by the *Plasmodium* sp. during its erythrocytic cycle [5]. *Plasmodium* sp. associated molecular patterns (PAMP) akin to glycosylphosphatidylinositol (GPI), various parasite proteins and the pro-oxidant malarial pigment (haemozoin) trigger the robust release of pro-inflammatory cytokines particularly tumour necrosis factor (TNF) and interleukin-1 (IL-1) by pattern recognition receptors [5, 7]. This pro-inflammatory cytokine profile (TNF and IL-1) sets in motion a cascade of events encompassing the recruitment of macrophages and inflammatory cells, nitric oxide (NO) production, alterations in vascular permeability, up regulation of adhesion molecules and microvascular accumulation of parasitized erythrocytes [7]. In the context of their T cell lineage, cytokines are classified as either Th1 or Th2 and the more recently delineated Th17 cytokine subset [8]. A reciprocal relationship exists between pro-inflammatory cytokines and their corresponding inflammation quelling (anti-inflammatory) counterparts. In general during acute malaria infection, the net effect of interactions between rapid pro-inflammatory Th1 type responses at the early stage(s) of infection generally mediated by IL-12, TNF, IL-6 and IFN-γ (necessary for parasite control) and robust timely anti-inflammatory Th-2 restricted responses mediated primarily by IL-10, IL-4 and TGF-β (vital for prevention of tissue damage) determine disease outcome [7, 9].

IL-35 has recently been elucidated as an anti-inflammatory member of the IL-12 family of heterodimeric cytokines [10]. Interleukin-35 is a heterodimer comprised of the α-chain of IL-12 (p35) linked to a β-chain constituted by the Epstein-Barr virus-induced gene 3 (EBI3) a subunit which it shares in common with IL-27 [11]. Immune modulating effects of IL-35 have been reported in several animal models of autoimmune disorders such as rheumatoid arthritis [12], diabetes mellitus [13], systemic lupus erythematosus [14], psoriasis [15] and a range of malignancies [16, 17]. Being principally secreted by regulatory T lymphocytes (Tregs), B cells and dendritic cells, IL-35 is believed to exert its biological action via the suppression of effector T cell proliferation, stimulation of B cell mediated IL-10 release and the propagation of a unique subset of IL-35 (iTr35) producing T cells [18–20]. Dendritic cells, plasma cells, and placental trophoblast cells represent additional cellular sources of IL-35 [19]. Signalling of IL-35 has been reported to occur via STAT 1 although signalling is speculated to occur via co-IL-12 family member receptors [19, 21]. Notwithstanding its dynamic roles reported in numerous models of human disorders, the characterization of immune response(s) involving IL-35 during malaria infection is yet to be elucidated. Consequently, this study aimed to characterize the expression profile and effect(s) of modulating IL-35 during *P. berghei* mouse model of malaria infection.

## Methods

### Reagents and kits

Solvents and reagents were procured from Sigma Aldrich (St. Louis, USA). Recombinant IL-35 protein (rIL-35) was supplied by Chimerigen Laboratories (Chimerigen, Liestal Switzerland), neutralizing anti-Epstein-Barr virus induced-gene 3 (AEBI3) antibody (clone V1.4C4.22) was supplied by Merk KGaA (Darmstadt, Germany). Mouse IL-35 sandwich ELISA kits were purchased from US biological (MA, USA). Mouse IL-35p35 antibody (catalogue no. MAB6688), immunoglobulin-G antibody (catalogue no. AF007) and anti-Rat Cell and Tissue staining kit (catalogue no. CTS017) were purchased from R&D systems (MN, USA).

### Experimental animals and ethics statement

Male ICR mice (initially weighing 17–20 g) aged between 4 and 5 weeks old were used for the animal study. The animals were procured from an authorized local supplier and housed in the animal house facility, Faculty of Medicine and Health Sciences, University Putra Malaysia. Mice were acclimatized for a period of 2 weeks on a standard 12 h light (0800–2000) and dark (2000–0800) cycle and maintained at constant room temperature (27 ^°^C) in adequately ventilated polypropylene cages (housing density of 3 mice per cage). Animals were granted unlimited access to normal rodent chow and clean water.

All animal handling procedures were performed in compliance with the guidelines revised and approved by the Institutional Animal Care and Use Committee of University Putra Malaysia (Approval No: UPM/IACUC/AUP-R018/2017). All tissue (blood) collection procedures were performed under anaesthesia with a mixture of ketamine (100 mg/kg) and xylazine (10 mg/kg) administered intraperitoneally.

### Parasite strain and animal infection

The rodent malaria parasite, *Plasmodium berghei* strain ANKA was acquired from the Institute of Medical Research (IMR), Kuala Lumpur Malaysia. Subsequent to one in vivo passage malaria infection was established in mice according to the methods previously described by Basir et al. [22]. The procedure involved obtaining blood via cardiac puncture under anaesthesia from a previously infected donor mouse. Blood obtained was diluted with normal saline such that 0.2 mL of the diluted blood contained approximately 2 × 10^7^ parasitized red blood cells (PRBC’s). Two hundred microlitre (200 µL) of the diluted blood was subsequently administered via intraperitoneal (i.p) injection to the group(s) of mice designated *P. berghei* infection group while the other group of mice representing the uninfected (control) group received 0.2 mL of similarly diluted uninfected blood obtained from a healthy donor mouse.

### Parasitaemia assessment

Parasitaemia was monitored via thin blood films from tail venesection prepared daily for each mouse by Wedge (two-slide) method on 25 × 75 mm glass slides. Blood films were stained with Leishman’s reagent and examined at 1000× total magnification under a light microscope (Olympus CX31). Specifically, five distinct microscopic fields for each slide (comprising approximately 200 cells per field) were enumerated via the crenellation technique [23]. Parasitaemia was enumerated as the percentage of total red blood cells that were parasitized in a minimum of five microscopic fields.

### Enzyme-linked immunosorbent assay

The concentration of IL-35 in systemic circulation was quantified in serum from *P. berghei* infected mice and uninfected mice (n = 5 mice/group) on day 1, 3 and 5 post *P. berghei* infection. At the designated time points, following the onset of anaesthesia (indicated by unresponsive toe reflex), whole blood (approximately 500 µL) was withdrawn via cardiac puncture from both *P. berghei* infected mice and their uninfected control counterparts. The experiment was performed twice (n = 10) for each time point assessed.

Serum separation was achieved by centrifugation of clotted blood at 1000 rpm for 15 min followed by subsequent storage at − 80 °C (Thermo Scientific, USA) pending further use. Serum samples were analysed in duplicate by enzyme linked immunosorbent assay (ELISA) using a quantikine mouse IL-35 ELISA kit (US biological MA, USA). Subsequent to the last enzyme reaction, plates were read with the aid of a micro plate reader (VersaMax^®^ Molecular Devices, China) at a wavelength of 450 nm.

### Immunohistochemistry procedure

Formalin fixed paraffin embedded tissues from *P. berghei* infected and uninfected (control) mice harvested on day 1, 3 and 5 following *P. berghei* infection were stained with monoclonal anti-IL-35p35 antibody aided by an anti-Rat HRP-DAB cell and tissue staining kit for the detection of IL-35p35 in sections from brain, liver, lung, spleen, heart and kidney.

Using a microtome (Leica RM 2255, Germany) 4 µm thick serial sections of each organ at 40 µm intervals were prepared on polysine coated glass slides (Thermo Scientific, USA). Air dried slides were dewaxed in an oven (Venticell MMM, Germany) set at 60 °C for 1 h and subsequently rehydrated prior to staining by passage through two changes of xylene and graded concentrations of ethanol using an automated tissue slide stainer (Tissue e-Tek Prisma, Japan). Subsequent to antigen retrieval, slides were depigmented to eliminate malarial pigment (haemozoin) by immersion in a saturated alcoholic solution of picric acid for 15 min [24, 25]. Heat induced epitope retrieval (HIER) was conducted with freshly prepared sodium citrate (plus tween 20) antigen retrieval buffer at pH 6.0 for 15 min in a microwave oven (Panasonic^®^, Japan) set at medium high temperature (~ 98 °C). Following antigen retrieval tissue sections were allowed to cool for 20 min at room temperature and subsequently rinsed with distilled water before commencing staining.

Anti-Rat HRP-DAB cell and tissue staining kit (CTS017, R&D systems, MN, USA) in conjunction with 15 µg/mL monoclonal mouse IL-35p35 antibody (R&D systems, MN, USA) incubated overnight at 4 °C (NR-C43TA, Nasional, Malaysia) were utilized for the immunohistochemistry procedure. Following overnight incubation, sections were subjected to a prolonged wash in phosphate buffered saline and incubated for 30 min with secondary antibody. After rinsing off excess secondary antibody, visualization of positive staining was achieved by incubating tissue sections with few drops of diaminobenzidine tetrahydrochloride (DAB) in a stabilizing buffer for 6 min followed by counter staining with Meyer’s haematoxylin for 15 s.

The stained tissue sections were dehydrated and cover slipped with DPX mountant. Images were acquired for each tissue section using a light microscope (CX31 Olympus, Germany) fitted with Cell F imaging software. The images acquired were analysed blindly by two independent pathologists.

### IL-35 modulation procedure

Following the assessment of IL-35 expression in systemic circulation and tissues during *P. berghei* infection, modulation of IL-35 was performed daily from the 1st day to the 4th day after *P. berghei* infection as follows;

Group 1 (uninfected): mice received daily intraperitoneal injections of sterile PBS.

Group 2 (PBS treated): infected mice received daily intraperitoneal injections of sterile PBS as the representative mock treated control to mice that received rIL-35 protein.

Group 3 (IgG antibody treated): infected mice received daily intraperitoneal injections of IgG antibody (15 µg/mL) as the representative mock treated control to mice treated with neutralizing AEBI3 antibody.

Group 4 (rIL-35 protein treated): infected mice received daily intraperitoneal injections of rIL-35 protein (5 µg/mL).

Group 5 (AEBI3 antibody treated): infected mice received daily intraperitoneal injections of neutralizing anti-Epstein–Barr virus induced-gene 3 antibody (25 µg/mL).

Doses of drugs administered were selected after consultation of related literature and results from preliminary studies. Serum for all mice was prepared from peripheral blood (approximately 500 µL) withdrawn by cardiac puncture performed on the 5th day after initiation of *P. berghei* infection.

### Flow cytometry analysis

To assess the impact of modulating IL-35 on the cytokine environment during *P. berghei* infection, cytometric bead array procedure for the simultaneous detection of IL-10, IL-17A, TNF, IFN-γ, IL-6, IL-4 and IL-2 was performed on serum samples collected from uninfected control mice and *P. berghei* infected mice on the 5th day following *P. berghei* infection after IL-35 modulation treatment. The assay was performed using a commercially procured mouse Th1/Th2/Th17 cytometric bead array kit (BD biosciences, San Jose, CA, USA). Staining procedure(s) were conducted as described (Instruction manual BD CBA Mouse Th1/Th2/Th17 cytokine kit, catalogue no. 560485). Sample acquisition was achieved using a BD LSR Fortessa fluorescence activated cell sorter via phycoerythrin (PE) and allophycocyanin (APC) channels. Prior to sample acquisition, gating layouts were generated for 2400 events from unstained control tubes and data acquired was analysed with FCAP Array software version 3.0 (BD Bioscience, San Jose, CA, USA).

### Histological assessment

On the 5th day following *P. berghei* infection, subsequent to IL-35 modulation treatment, harvested organ specimens were fixed with 10% neutral buffered formalin, processed and embedded in paraffin blocks. Slides bearing 4 µm thick sections of each organ specimen from the various groups were subjected to haematoxylin and eosin staining per routine histology procedures in an automated slide stainer (e-Tek Prisma, Japan). Stained tissue sections were dehydrated and mounted with DPX-mountant prior to visualization aided by a light microscope (CX31 Olympus, Germany) fitted with Cell F imaging software. Blind examination of the stained slides was carried out independently by two pathologists. Evidence of pathological changes typical of malaria infection including PRBC sequestration, tissue inflammation, architectural loss and malarial pigment deposition were categorized on a score from 0 to 3 as follows; Score 0 = “normal tissue” without evidence of PRBC sequestration, tissue inflammation, architectural loss or malarial pigment deposition, Score 1 = evidence of pathological features consistent with malaria infection in less than 25% of the field of view, Score 2 = evidence of pathological features consistent with malaria infection in 25 to 50% of the field of view and Score 3 = evidence of pathological features consistent with malaria infection in greater than 50% of the field of view.

### Assessment of survival

Following the modulation of IL-35 in groups of *P. berghei* infected mice, percentage survival of mice during the course of the infection was assessed daily. Incidence(s) of mortality observed per day was recorded as the number of dead mice in a particular group out of the total number of mice comprising the group multiplied by 100%.

### Statistical analysis

Statistical analyses were performed using GraphPad Prism software (Prism 7, GraphPad Software, Inc., CA, USA). Significant differences between treatment groups were analysed using one-way Analysis of Variance (ANOVA) followed by Tukey’s (honestly significant difference) multiple comparisons as post hoc test. Correlation between serum IL-35 and parasitaemia development was deduced with the aid of linear regression analysis while Pearson’s correlation analysis was used to illustrate correlationship between treatment groups. A probability value of less than 0.05 (*p *< 0.05) was considered to be statistically significant.

## Results

### IL-35 was up regulated in serum and correlated with parasitaemia levels during *P. berghei* infection

**Fig. 1:**
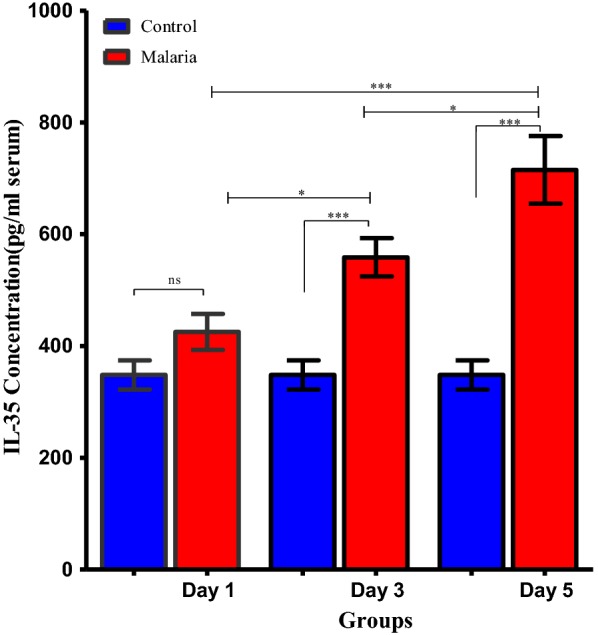
Serum IL-35 concentration measured in *P. berghei* infected and uninfected mice. One-way ANOVA between groups revealed significant elevation of serum IL-35 concentration as *P. berghei* infection progressed [F (5, 55) = 17.21; **p < 0.0001; r^2^ = 0.608], n = 10. Results are expressed as mean ± sem. Data shown is a representative from two experiments

**Fig. 2:**
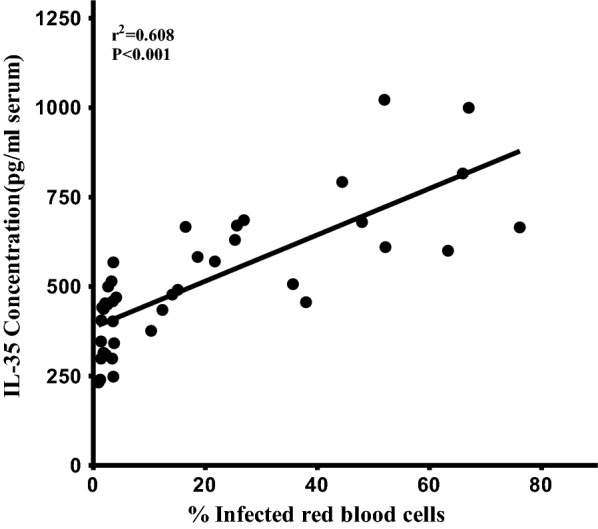
Scatter plot showing a positive correlationship between serum IL-35 concentration (pg/mL) and percentage parasitaemia development in *P. berghei* infected mice, n = 10, (r^2^ = 0.608, ***p < 0.0001). Pearson’s correlation coefficient was r = 0.780. All results are expressed as mean ± sem. Data shown is a representative from two experiments

Assessment of IL-35 concentration at different time intervals during the course of *P. berghei* infection by ELISA revealed that serum IL-35 concentration during the course of malaria infection was significantly elevated in *P. berghei* infected mice compared to uninfected control mice (Fig. 1). Specifically, expression of IL-35 increased with increasing parasitaemia as *P. berghei* infection progressed. Elevated serum concentration of IL-35 was particularly evident on the 3rd day following *P. berghei* infection at 558.6 ± 34.34 pg/mL and on the 5th day post *P. berghei* infection at 715.1 ± 60.51 pg/mL when compared to *P. berghei* infected mice on the 1st day post infection with serum IL-35 concentration of 425.2 ± 32.05 pg/mL and uninfected controls with serum IL-35 concentration of approximately 348.1 ± 26.12 pg/ml, 336 ± 22.51 pg/ml and 345 ± 20.52 pg/mL on day 1, 3 and 5, respectively. Serum IL-35 concentration was found to correlate positively and significantly (r^2^ = 0.608, *p *< 0.001) with increasing parasitaemia (Fig. 2). The elevated serum IL-35 concentration indicates that IL-35 is produced during *P. berghei* infection and its production correlates with parasitaemia progression.

### IL-35 is expressed in tissues of *P. berghei* infected mice

**Fig. 3:**
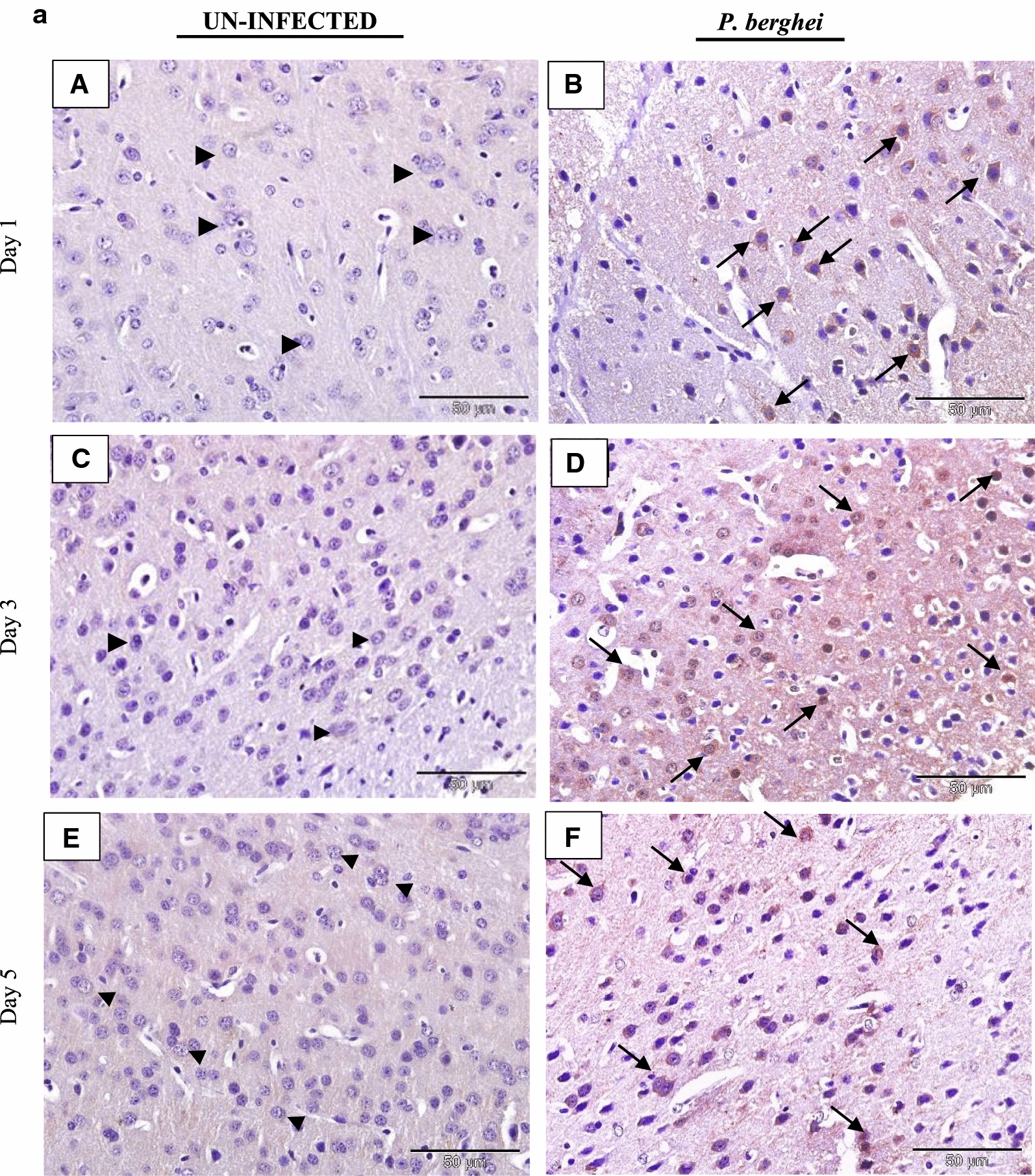

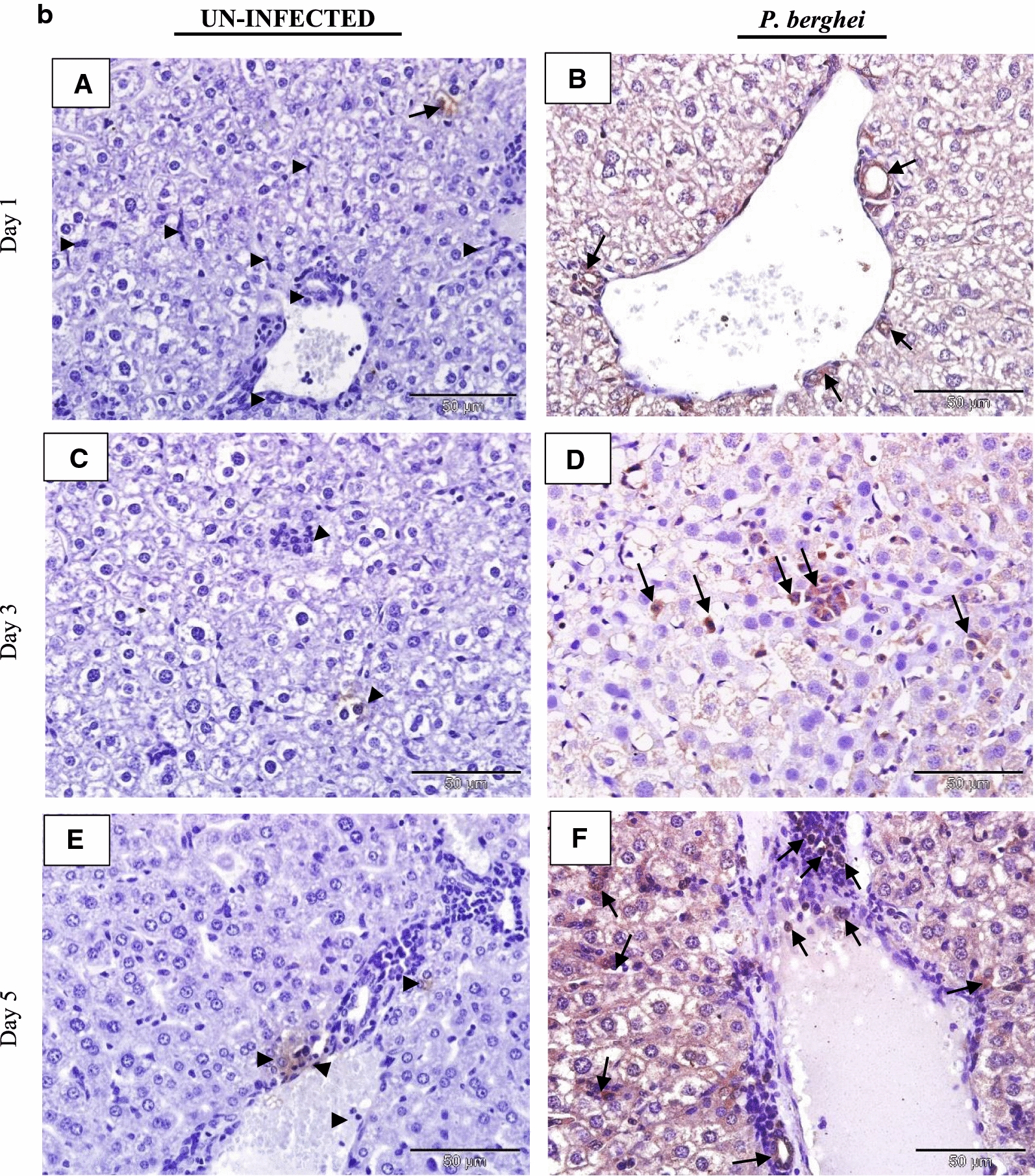

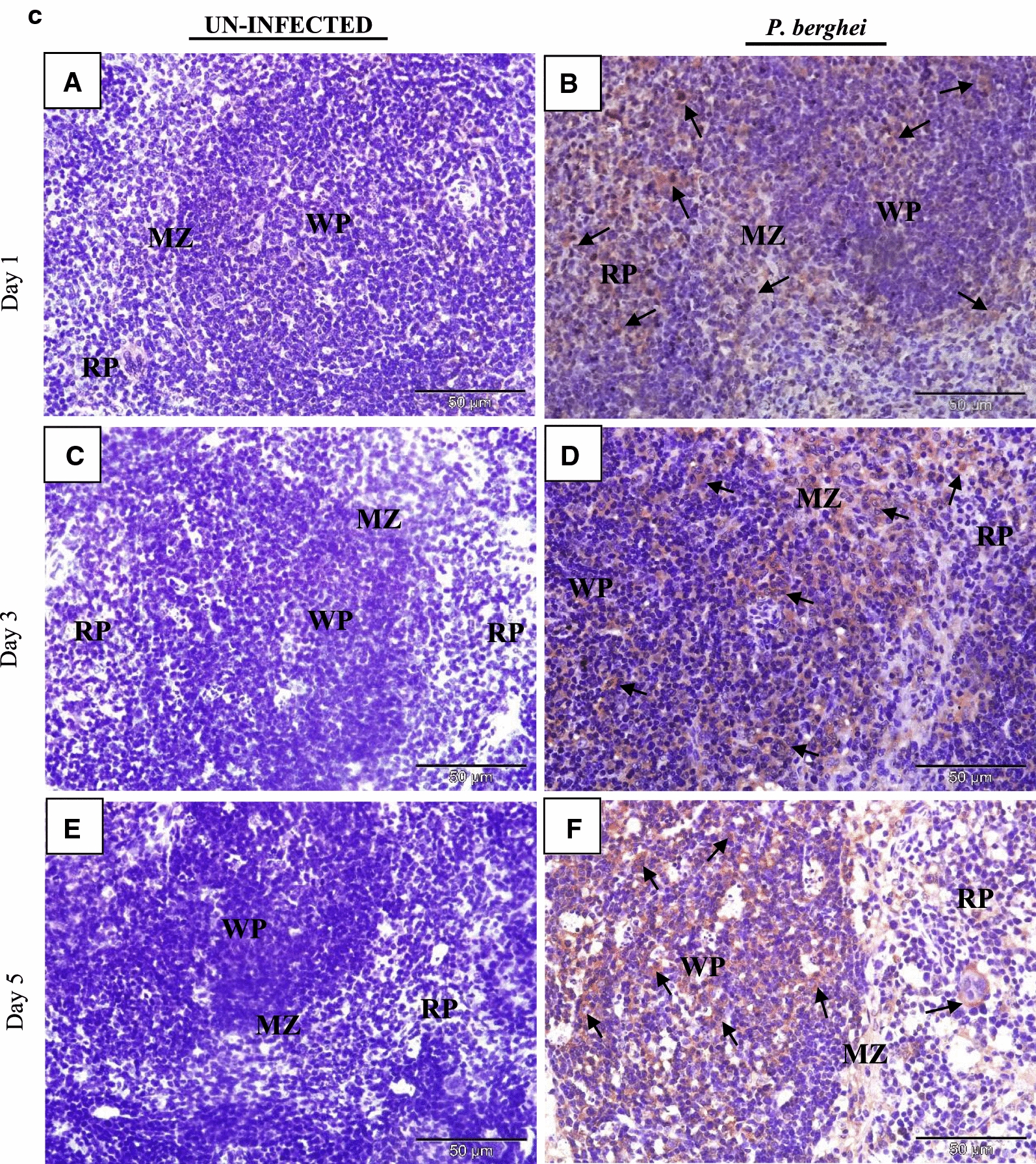

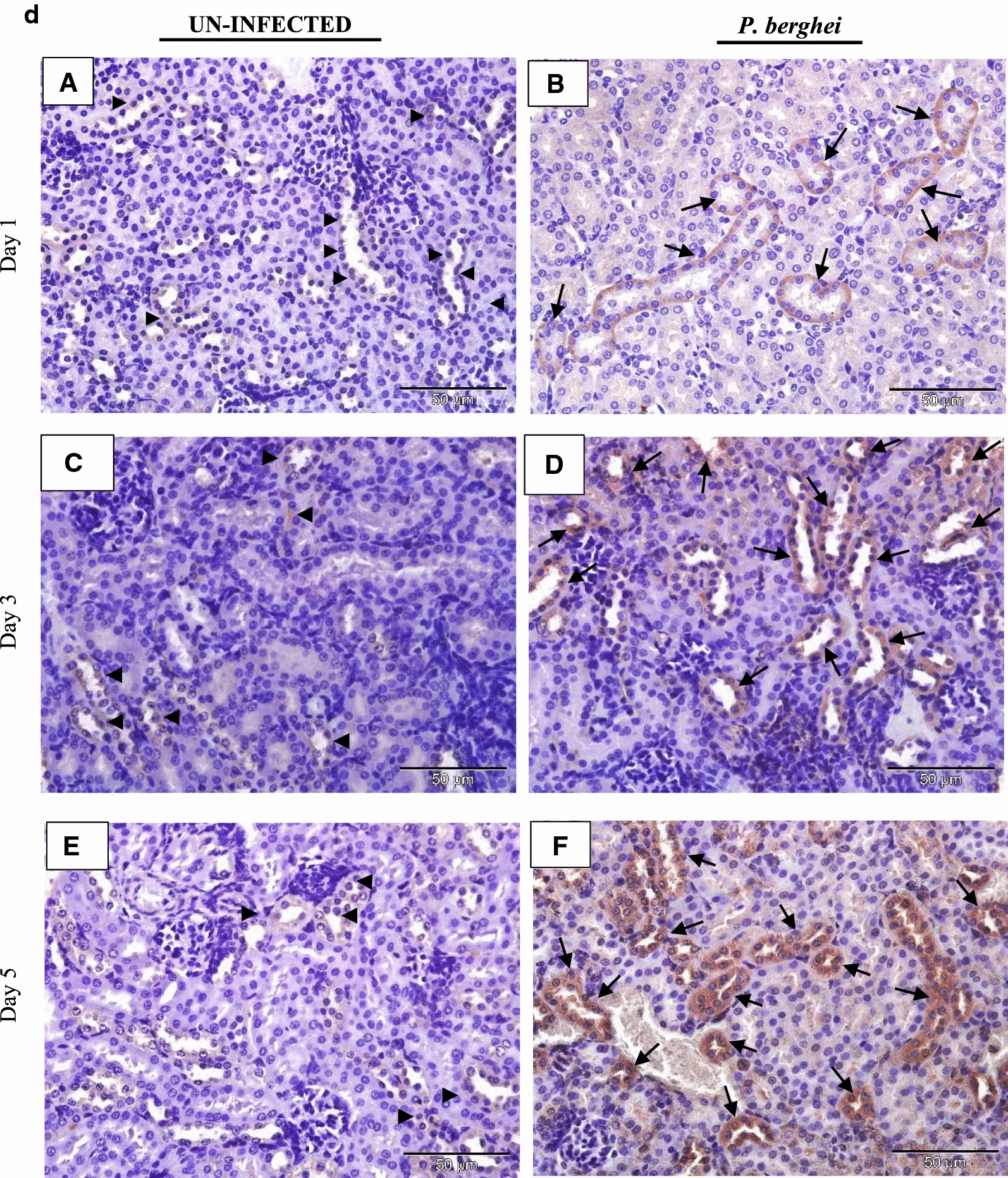

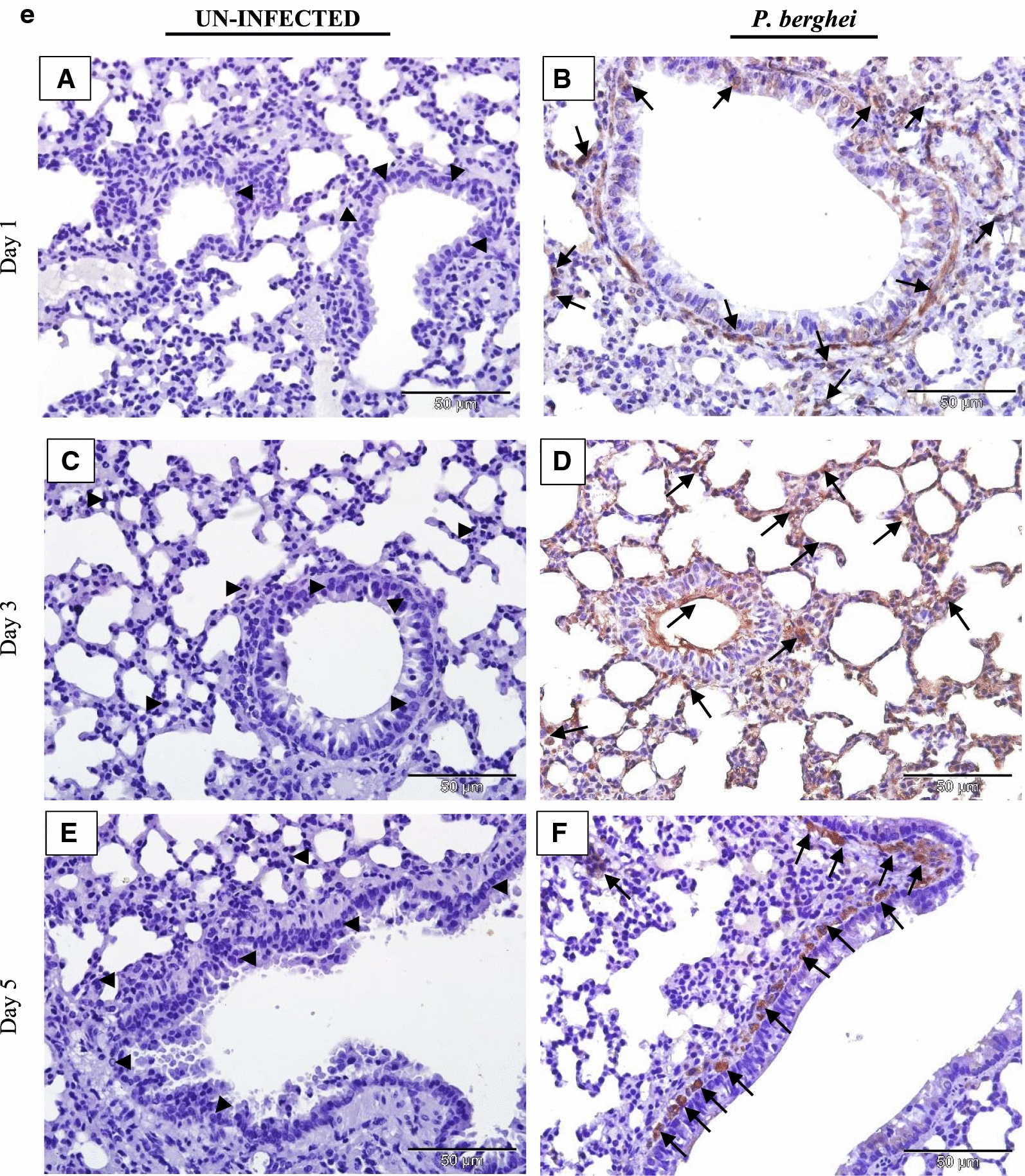

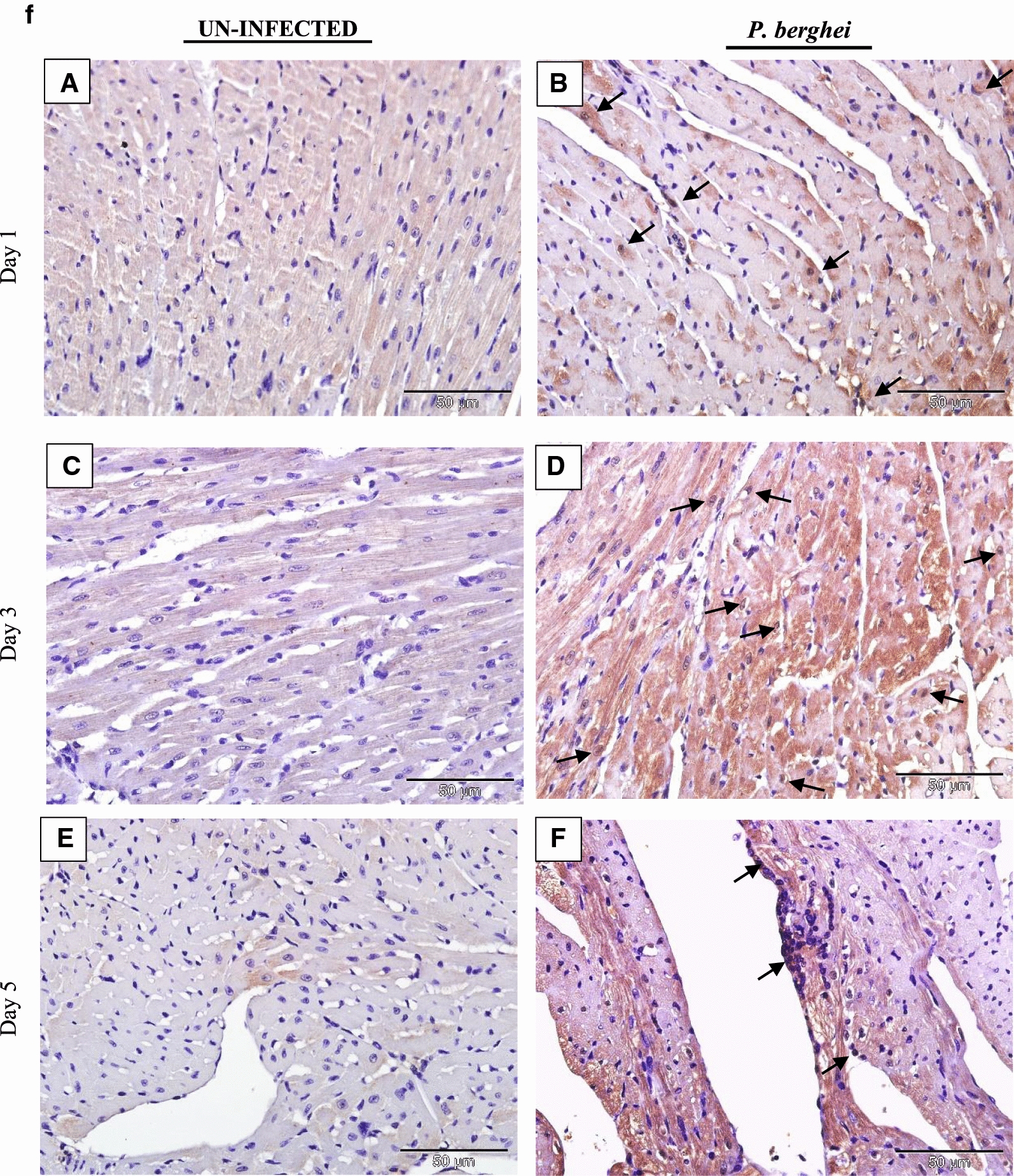
**a** Immunohistochemical staining showing the localization of IL-35p35 in mouse brain. Each panel is a representative photomicrograph from either 5 control mice (**A**, **C**, **E**) or 5 *P. berghei* infected mice (**B**, **D**, **F**). In *P. berghei* infected mouse brain (**B**, **D**, **F**) positive cytoplasmic staining (black arrows) was evident in the neurons of the cerebral cortex. Scale bar: 50 µm at ×400 total magnification. There was no immunoreactivity observed in the neurons of the cerebral cortex from uninfected mice (arrow heads). The signal was developed using HRP-labelled secondary antibody and DAB reagent, nuclei were counterstained with Meyer’s haematoxylin. Images shown are a representative from three experiments. **b** Immunohistochemical staining showing the localization of IL-35p35 in mouse liver. Each panel is a representative photomicrograph from either 5 control mice (**A**, **C**, **E**) or 5 *P. berghei* infected mice (**B**, **D**, **F**). In *P. berghei* infected mouse liver (**B**, **D**, **F**) positive cytoplasmic staining (black arrows) was evident in stellate macrophages (Kupffer cells) and epithelial cells lining the portal tract. Scale bar: 50 µm at ×400 total magnification. Marginal (constitutive) immunoreactivity was observed in uninfected mouse liver (arrow heads). The signal was developed using DAB reagent, nuclei were counterstained with Meyer’s haematoxylin. Images shown are a representative from three experiments. **c** Immunohistochemical staining showing the localization of IL-35p35 in mouse spleen. Each panel is a representative photomicrograph from either 5 control mice (**A**, **C**, **E**) or 5 *P. berghei* infected mice (**B**, **D**, **F**). In *P. berghei* infected mouse spleen (**B**, **D**, **F**) positive cytoplasmic staining (black arrows) was evident in a subset of splenocytes in spleen from *P. berghei* infected mice. Representative images were acquired at ×400 total magnification. Scale bar = 50 µm. Marginal (constitutive) immunoreactivity was observed in uninfected mouse spleen. The signal was developed using HRP-labelled secondary antibody and DAB reagent, nuclei were counterstained with Meyer’s haematoxylin. *WP* white pulp, *RP* red pulp, *MZ* marginal zone. Images shown are a representative from three experiments. **d** Immunohistochemical staining showing the localization of IL-35p35 in mouse kidney. Each panel is a representative photomicrograph from either 5 control mice (**A**, **C**, **E**) or 5 *P. berghei* infected mice (**B**, **D**, **F**). In *P. berghei* infected mouse kidney (**B**, **D**, **F**) positive cytoplasmic staining (black arrows) was evident in the renal cortex. Diffused membrane staining (immunoreactivity) was specifically localized to the renal tubules and intercalated cells of the collecting ducts. Scale bar: 50 µm at ×400 total magnification. Marginal constitutive immunoreactivity was observed in uninfected mouse kidney (arrow heads). Images shown are a representative from three experiments. **e** Immunohistochemical staining showing the localization of IL-35p35 in mouse lung. Each panel is a representative photomicrograph from either 5 control mice (**A**, **C**, **E**) or 5 *P. berghei* infected mice (**B**, **D**, **F**). In *P. berghei* infected mouse lung (**B**, **D**, **F**) positive cytoplasmic staining (black arrows) was evident in the epithelial cells lining the bronchioles, alveolar epithelium and epithelioid histiocytes (macrophages). Scale bar: 50 µm at ×400 total magnification. There was no immunoreactivity observed in uninfected mouse lung (arrow heads). The signal was developed using HRP-labelled secondary antibody and DAB reagent, nuclei were counterstained with Meyer’s haematoxylin. Images shown are a representative from three experiments. **f** Immunohistochemical staining showing the localization of IL-35p35 in mouse heart. Each panel is a representative photomicrograph from either 5 control mice (**A**, **C**, **E**) or 5 *P. berghei* infected mice (**B**, **D**, **F**). Scale bar: 50 µm at ×400 total magnification. The signal was developed using HRP-labelled secondary antibody and DAB reagent, nuclei were counterstained with Meyer’s haematoxylin. Images shown are a representative from three experiments

The tissue expression profiles of IL-35p35 during the course of *P. berghei* infection were assessed by immunohistochemistry. Comparisons were made between *P. berghei* infected mouse tissues harvested at specific intervals of time (day 1, 3 and 5) after initiation of *P. berghei* infection to similarly harvested uninfected control mouse tissues. All *P. berghei* infected mouse tissues assessed demonstrated varying intensities of immune reactivity for IL-35p35 possibly indicating the presence of IL-35 unlike similarly stained uninfected mouse tissues where marginal (constitutive) immunostaining for IL-35p35 was occasionally evident. In *P. berghei* infected mouse tissues, positive staining was observed as dense brown immunolabeling localized primarily to the cytoplasm of splenocytes in the follicular mantle zone, periarteriolar lymphocytic sheath (PALS) and marginal zones of the spleen. Neurons of the cerebral cortex in the brain, epithelial cells lining the portal tract and stellate macrophages of the liver were also positive for IL-35. Furthermore, alveolar macrophages and epithelial cells lining alveolar of the lung and renal tubules in addition to intercalated cells of the medullary collecting ducts in *P. berghei* infected mice were positive for IL-35p35 possibly indicating the presence of IL-35. Infected mouse spleen tissue was utilized as (internal) positive control for the experiment. The intensity of expression was noticeably increased with increasing parasitaemia as *P. berghei* infection progressed in sections from the kidney, lung and heart of *P. berghei* infected mice (Fig. 3d–f).

### Effect of modulating IL-35 on parasitaemia development during *P. berghei* infection

**Fig. 4:**
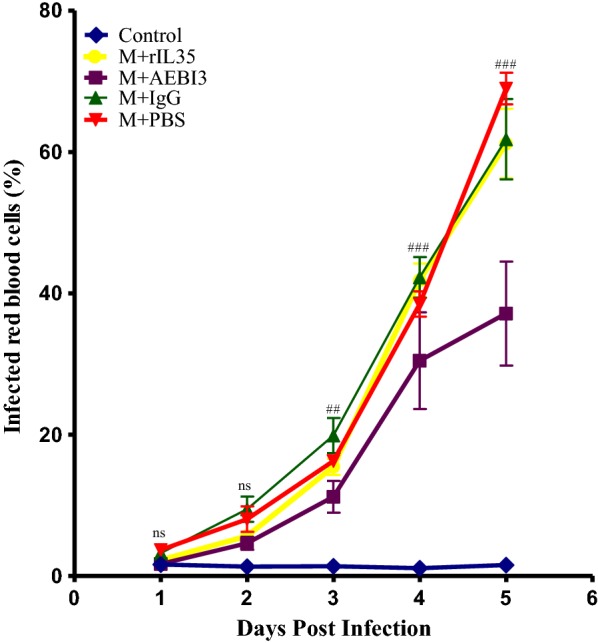
Comparative mean parasitaemia curves for uninfected control mice and *P. berghei* infected mice that received either rIL-35, neutralizing AEBI3 antibody, IgG antibody or PBS. *ns* not significant; ^#^comparison between IgG antibody and neutralizing AEBI3 treated mice. One symbol represents *p *< 0.05; two symbols represent *p *< 0.01; three symbols represent *p *< 0.001. One way ANOVA was used to analyse differences between treatment groups with Tukeys HSD as post hoc test. Squares, (inverted) triangles, circles and rhombi represent mean ± sem (n = 6). Data shown is a representative from two experiments

Comparative mean parasitaemia percentages expressed as percentage infected red blood cells for uninfected control mice and *P. berghei* infected mice that received either rIL-35 protein, neutralizing AEBI3 antibody, IgG antibody or sterile PBS treatment were as shown (Fig. 4). Mean parasitaemia percentage of *P. berghei* infected mice that received sterile PBS or rIL-35 protein were comparable while neutralizing AEBI3 treated mice demonstrated significantly lower parasitaemia percentages compared to their IgG antibody treated counterparts as shown in Fig. 4.

### Effects of modulating IL-35 on histopathological features manifested during malaria infection

**Fig. 5:**
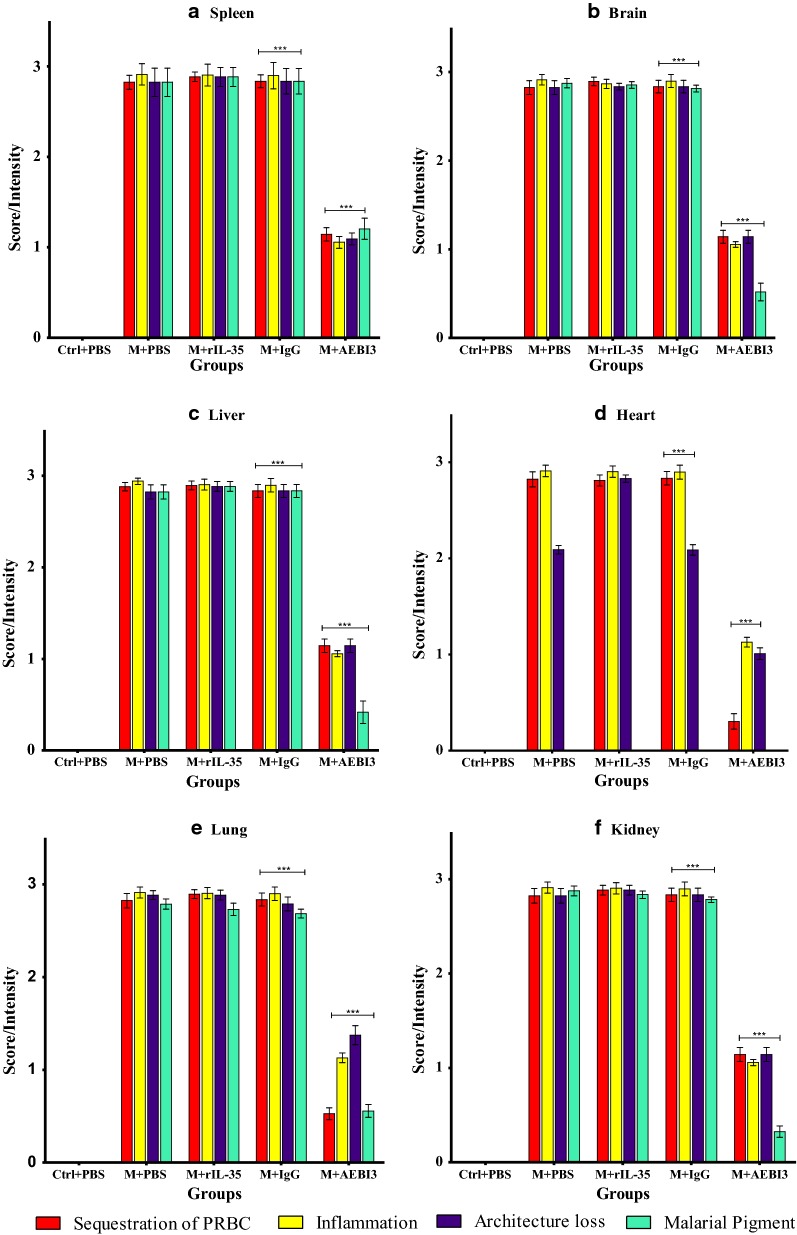
Histological scores of mouse spleen (**a**), brain (**b**), liver (**c**), heart (**d**), lung (**e**) and kidney (**f**) in uninfected mice and *P. berghei* infected mice following IL-35 modulation. Ctrl + PBS = uninfected + Phosphate buffered saline; M + PBS = *P. berghei* + Phosphate buffered saline; M + IgG = *P. berghei* + immunoglobulin G antibody; M + rIL-35 = *P. berghei* + recombinant IL-35 protein; M + AEBI3 = *P. berghei* + neutralizing anti Epstein Barr virus induced protein-3 antibody. Significant differences in histological scores (**p < 0.001) were observed between infected mice treated with neutralizing AEBI3 antibody and mice treated with IgG antibody. Data are mean ± sem (n = 6), Data shown is a representative from three experiments

Histological examination of organs harvested from infected mice that received recombinant IL-35 protein as well as their PBS-treated control counterparts and IgG antibody treated mice revealed features consistent with severe malaria infection. Overall, PBS-treated and rIL-35 protein treated mice demonstrated similar tissue pathology. However, mice treated with neutralizing AEBI3 antibody demonstrated relatively normal histology in most organs when compared to infected mice treated with IgG antibody. The appropriation of histological scores was carried out upon microscopic assessment of respective tissue sections from uninfected mice and *P. berghei* infected mice that received either PBS, IgG antibody, rIL-35 protein or neutralizing AEBI3 antibody (n = 6 mice/group). Histological scores were appropriated on the basis of four major histopathological features associated with malaria infection namely; PRBC sequestration in microvessels, accumulation of malarial pigment (haemozoin), inflammation and architectural loss evidenced by the distortion of regular tissue architecture (Fig. 5).

**Fig. 6:**
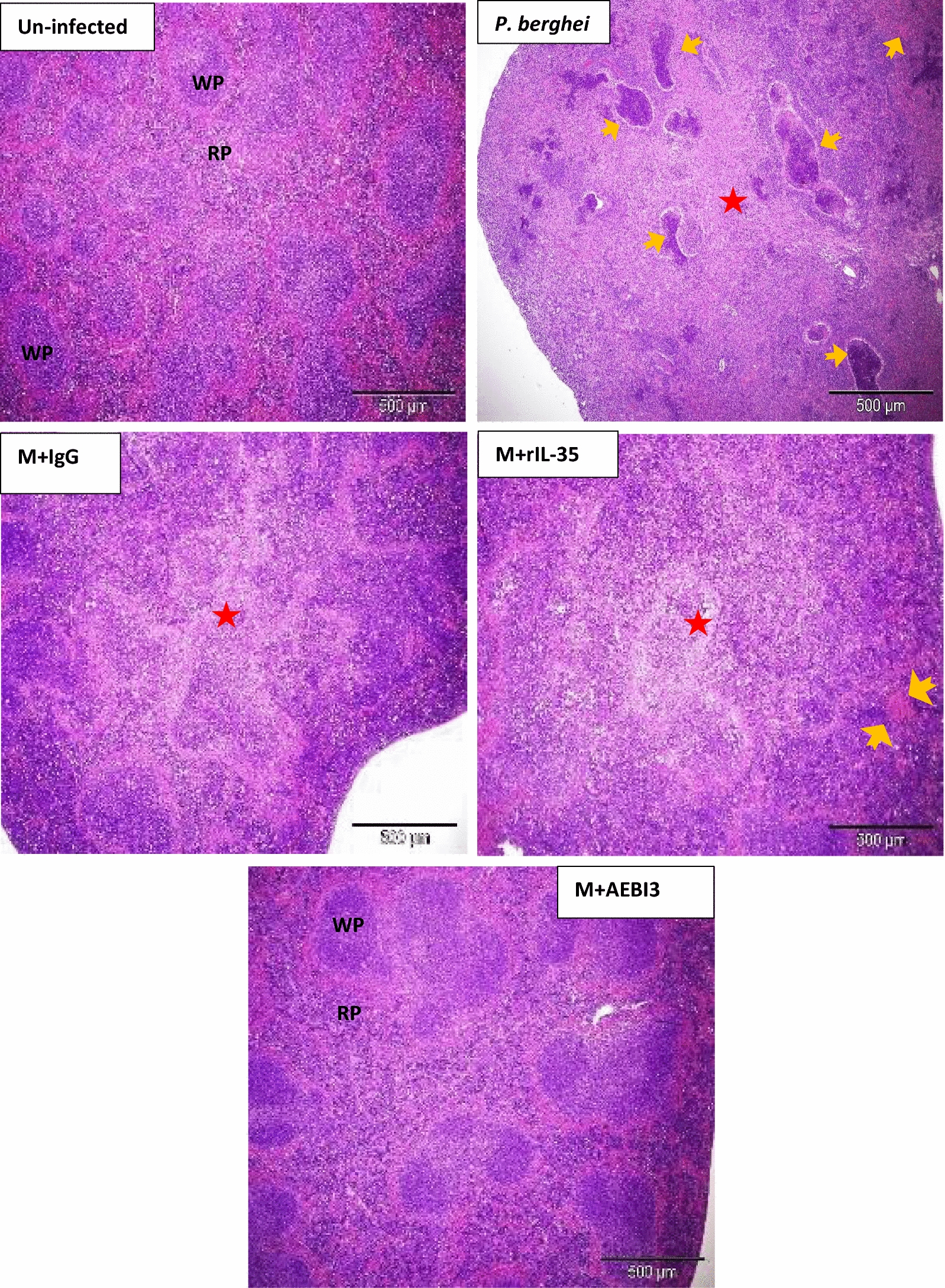
Microscopic observations of H&E staining on day 5 post *P. berghei* infection in the spleen of an uninfected control mouse and *P. berghei* infected mice following the modulation of IL-35. Uninfected mouse spleen showed distinct regions of red (RP) and white (WP) pulp. Note the presence of splenic infarction (yellow arrows) and loss of central germinal structure (red star). Images were acquired at ×40 total magnification (scale bar; 500 µm Photomicrographs are representative images from three experiments

**Fig. 7:**
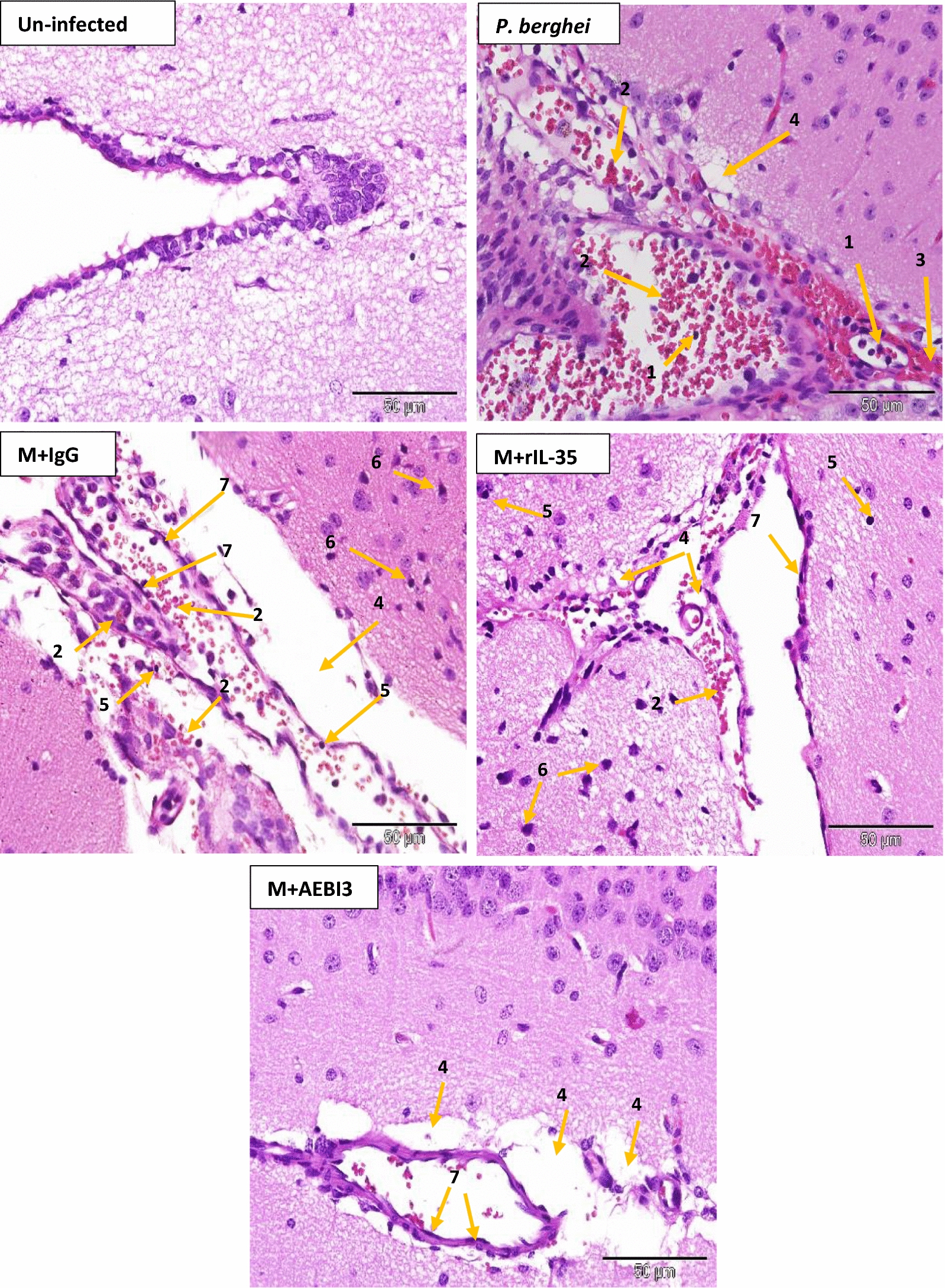
Microscopic observations of H&E staining on day 5 post *P. berghei* infection in the brain of an uninfected control mouse and *P. berghei* infected mice following the modulation of IL-35. Note the presence of intravascular lymphocytes (1), PRBC sequestration and formation of rosettes (2), perivascular haemorrhage (3), perivascular space enlargement (4), lymphocytes (5), pyknotic neurons (6) and pericytes (7). Images were acquired at ×400 total magnification (Scale bar; 50 µm). Photomicrographs are representative images from three experiments

Histological assessment of sections from the spleen of infected mice demonstrated hyperplasia of the red and white pulps with an associated loss of central germinal structure. Thickening of splenic trabeculae, congestion of splenic cords with malarial pigment (haemozoin) laden macrophages in addition to focal areas of haemorrhage and infarction were also observed (Fig. 6). Histological examination of sections from the brain of *P. berghei* infected mice treated with sterile PBS, rIL-35 protein or IgG antibody (Fig. 7) revealed variable presence of large numbers of PRBCs sequestered in small cerebral capillaries and venules in addition to haemozoin pigment deposition, perivascular haemorrhages, oedema and proliferation of microglial cells.

**Fig. 8:**
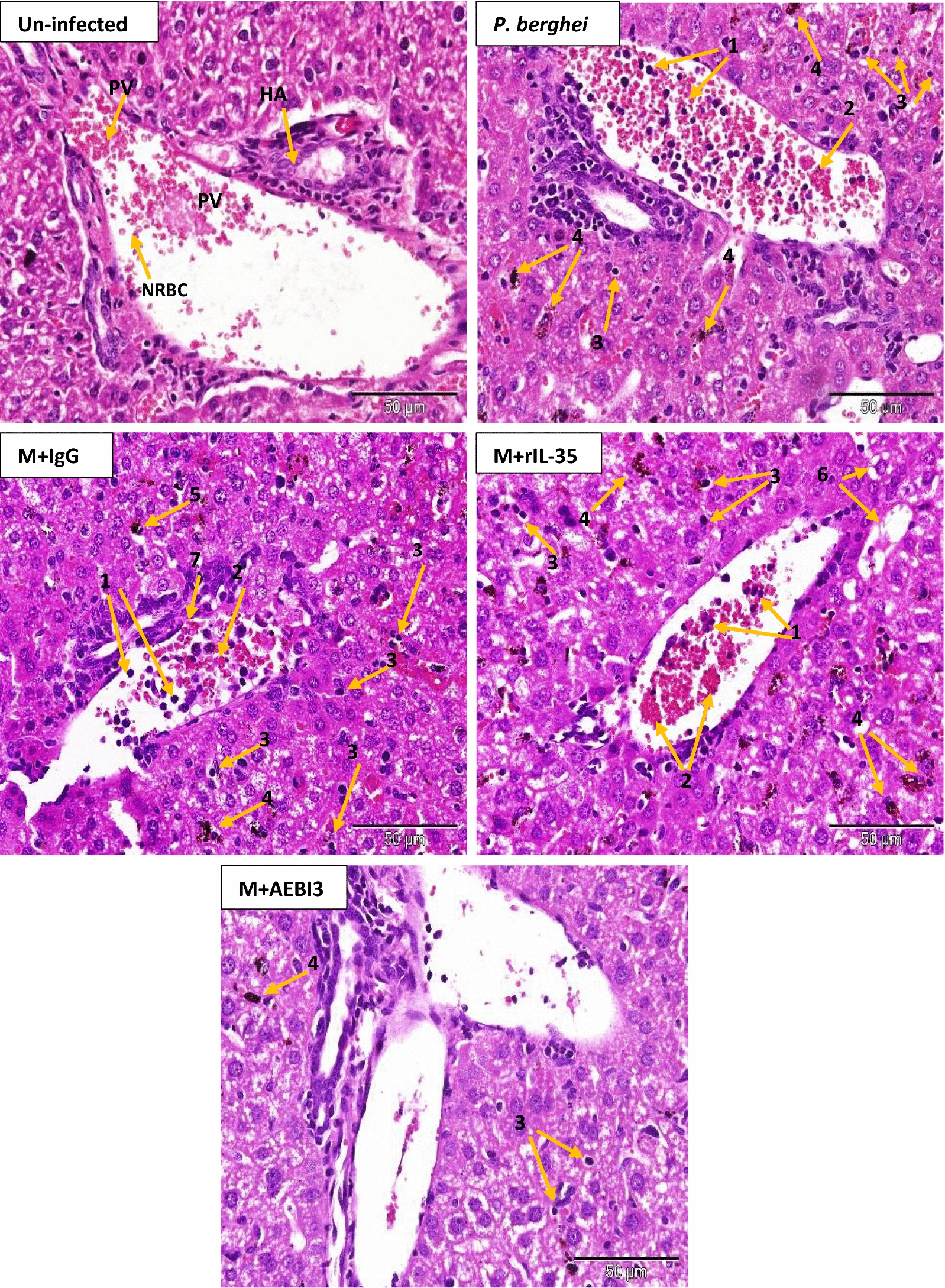
Microscopic observations of H&E staining on day 5 post *P. berghei* infection in the liver of an uninfected control mouse and *P. berghei* infected mice following the modulation of IL-35. Uninfected mouse liver showing hepatic artery (HA), portal vein (PV) and normal red blood cells (NRBC). The portal vein in sections from infected mice was congested with PRBC’s, macrophages and lymphocytes (1) in addition to rosettes of PRBC’s (2). Hyperplasia and hypertrophy of stellate macrophages/von kupffer cells (3), haemozoin pigment deposition in liver parenchyma (4), sinusoidal dilatation and congestion with malarial pigment (haemozoin) and PRBC’s (5), hydropic degeneration (6), PRBC sequestration and adherence to endothelial wall (7) were also apparent. Images were acquired at ×400 total magnification (scale bar; 50 µm). Photomicrographs are representative images from three experiments

**Fig. 9:**
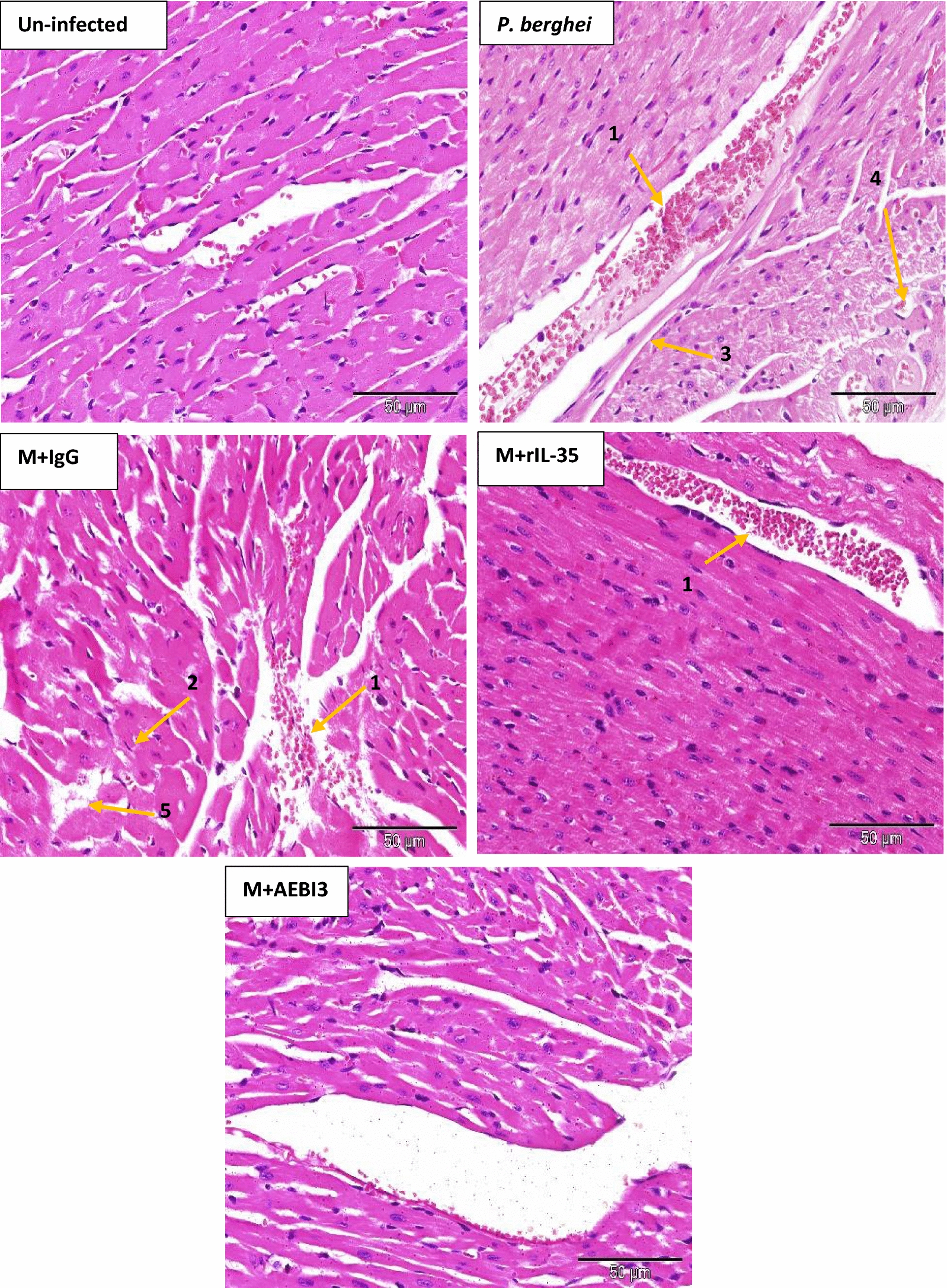
Microscopic observations of H&E staining in uninfected and *P. berghei* infected mouse heart on day 5 post *P. berghei* infection following the modulation of IL-35. Note the congestion of cardiac blood vessels with PRBC’s, lymphocytes and macrophage engulfing elements (1), presence of cardiac myocytes (2), perivascular oedema (3), vacuolar degeneration (4) and interstitial oedema (5). Images were acquired at ×400 total magnification (Scale bar; 50 µm). Photomicrographs are representative images from three experiments

**Fig. 10:**
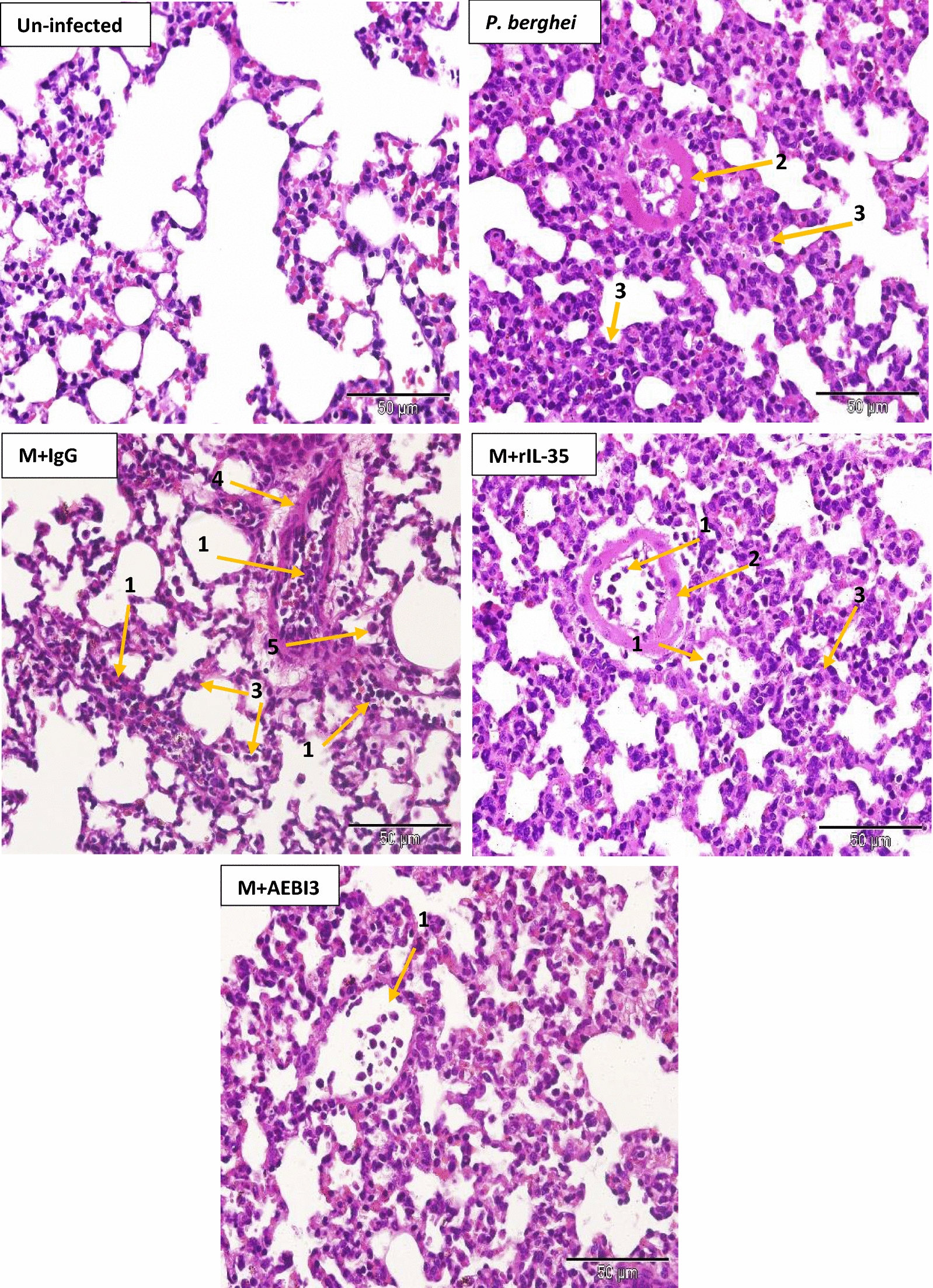
Microscopic observations of H&E staining on day 5 post *P. berghei* infection in the lung of an uninfected mouse and *P. berghei* infected mice following the modulation of IL-35. Note presence of PRBC’s, lymphocytes and macrophages in pulmonary vessel (1), hyaline membrane formation (2), thickening of the alveolar septae (3), hyalinised blood vessel (4) and macrophages (5). Images were acquired at ×400 total magnification (scale bar; 50 µm). Photomicrographs are representative images from three experiments

**Fig. 11:**
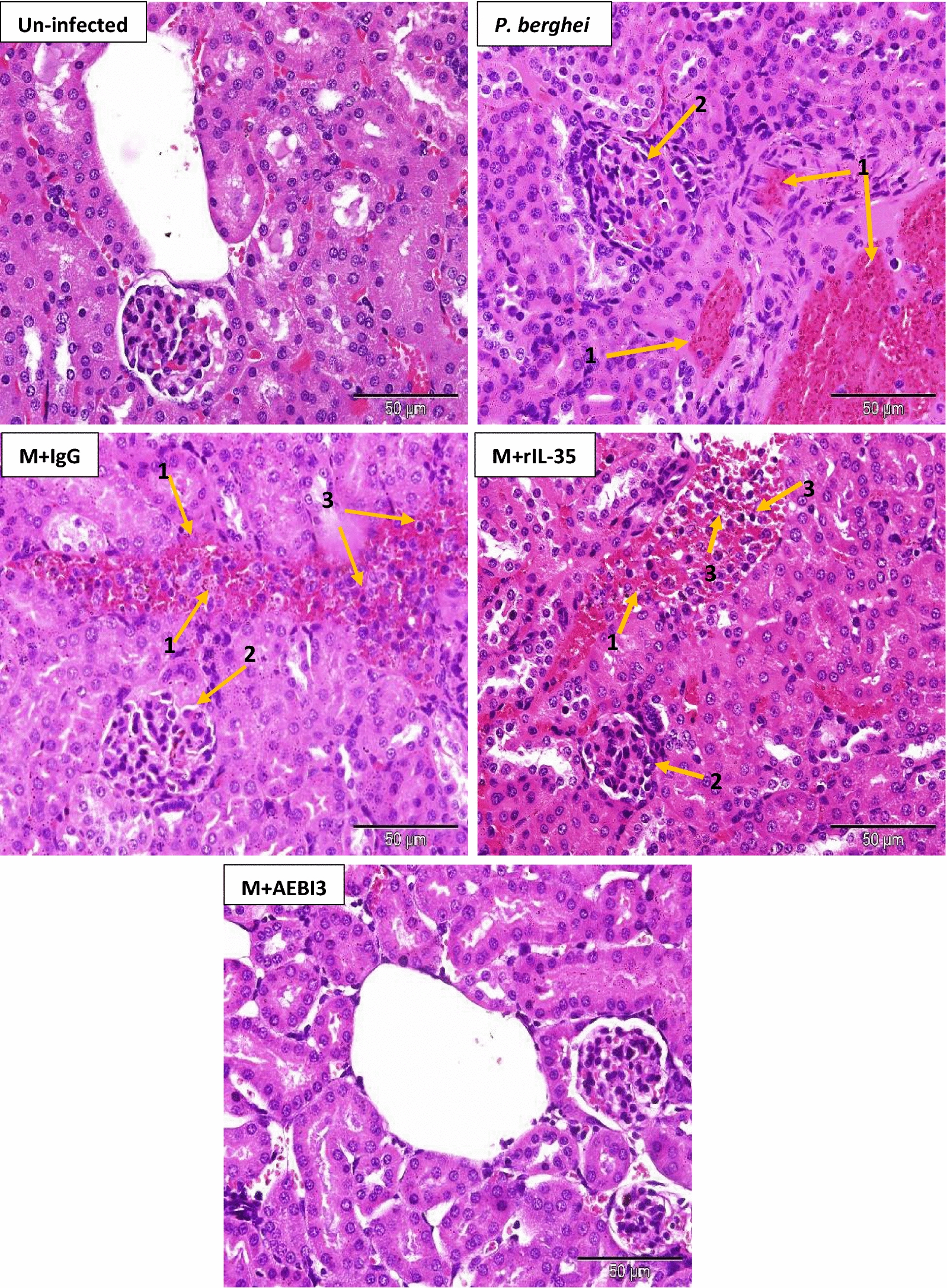
Microscopic observations of H&E staining on day 5 post *P. berghei* infection in kidney sections from an uninfected control mouse and *P. berghei* infected mice following the modulation of IL-35. Evidence of haemorrhage (1), loss of structure of the malpighian corpuscule and hypercellularity of the glomerulus (2) in addition to renal vascular accumulation of PRBC’s (3) were apparent. Images were acquired at ×400 total magnification (scale bar; 50 µm). Photomicrographs are representative images from three experiments

Similarly, examination of sections from the liver of *P. berghei* infected mice that received either PBS, rIL-35 protein or IgG antibody (Fig. 8) revealed dilated hepatic sinusoids densely congested with parasite pigments (haemozoin), while the portal tract was congested with large numbers of parasitized erythrocytes and haemozoin crystals. Histological examination of cardiac tissue (Fig. 9) demonstrated accumulation of parasitized erythrocytes within cardiac capillaries predominantly in cardiac sections obtained from *P. berghei* infected mice treated with either sterile PBS, rIL-35 protein or IgG antibody. In addition to the accumulation of parasitized erythrocytes, further features observed in cardiac tissue sections from infected mice included the appearance of discontinuous cardiac muscle fibres, resident cardiac macrophages, intramuscular haemorrhage, vacuolar degeneration and perivascular oedema. Sections of the lung tissue obtained from *P. berghei* infected mice (Fig. 10) revealed congestion of alveolar septae with PRBC’s in addition to hyaline membrane formation. Pathological manifestations consistent with malaria infection in sections of renal tissue from *P. berghei* infected mice (Fig. 11) were inclusive of increased glomerular cellularity, as well as the accumulation of PRBC’s, leukocytes and macrophages in the renal interstitium.

### Effects of modulating IL-35 on cytokine release during *P. berghei* infection

**Fig. 12:**
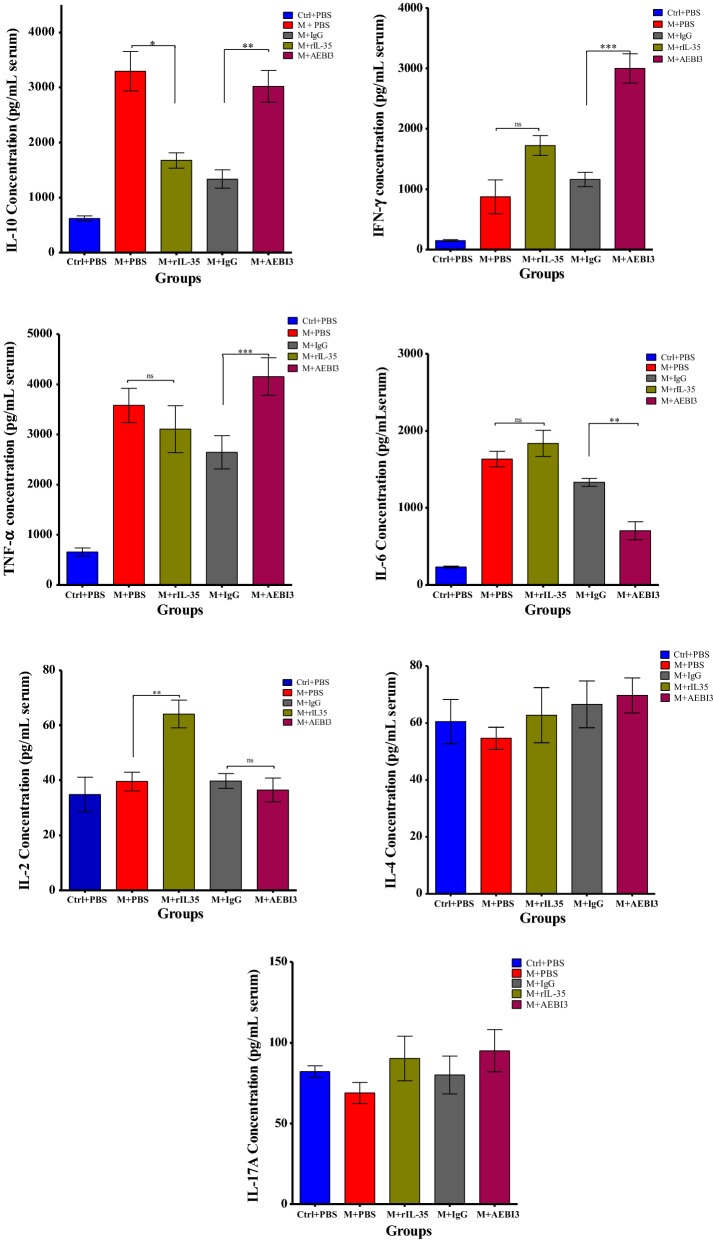
Effect of IL-35 modulation on inflammatory cytokine profiles of *P. berghei* infected mice on day 5 post *P. berghei* infection. Ctrl + PBS = uninfected + phosphate buffered saline; M + PBS = *P. berghei* + phosphate buffered saline; M + IgG = *P. berghei* + immunoglobulin G antibody; M + rIL-35 = *P. berghei* + recombinant IL-35 protein; M + AEBI3 = *P. berghei* + neutralizing anti Epstein Barr virus induced protein-3 antibody. Data are mean ± sem (n = 6). Data shown is a representative from two experiments

Generally, *P. berghei* infected mice that received recombinant IL-35 protein in comparison to their PBS treated counterparts demonstrated similar levels of TNF, IFN-γ and IL-6, elevated serum levels of IL-2 (*p *< 0.01) and decreased serum levels of IL-10 (*p *< 0.05). Infected mice that received neutralizing AEBI3 antibody treatment demonstrated markedly increased serum levels of IFN-γ (***p < 0.001) and lower levels of IL-6 (**p < 0.01) compared to IgG antibody treated mice. Additionally IL-10 and TNF levels in *P. berghei* infected mice that received neutralizing AEBI3 antibody were also elevated in comparison to levels demonstrated by IgG antibody treated mice (*p *< 0.01; *p *< 0.001 respectively). Serum levels of IL-4 and IL-17 were not significantly different between the treatment groups assessed (Fig. 12).

### Effect of modulating IL-35 on survival of *P. berghei* infected mice

**Fig. 13:**
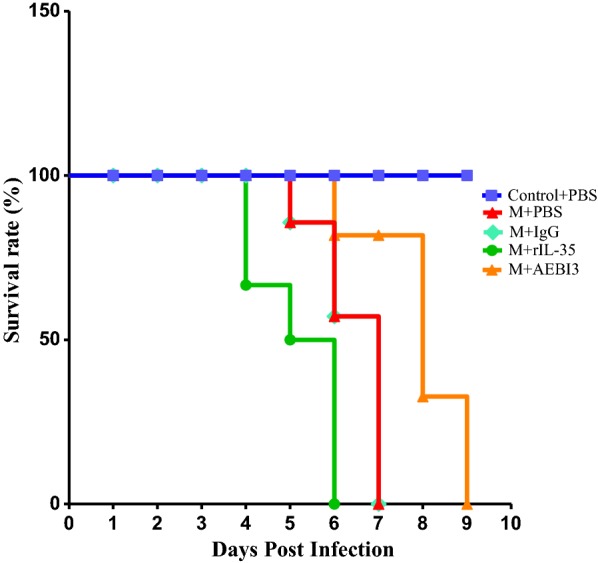
Kaplan Meier curve depicting the effects of modulating IL-35 on survival of *P. berghei* infected and uninfected ICR mice. Death was observed and recorded daily. Overall, mice that received neutralizing AEBI3 antibody treatment demonstrated prolonged survival compared to IgG antibody treated mice. Data shown is a representative from two experiments

Recombinant IL-35 treated mice showed a tendency towards early mortality beginning by day 4 post infection with *P. berghei* compared to PBS treated mice. The group of *P. berghei* infected mice to which neutralizing AEBI3 antibody was administered demonstrated prolonged survival and only succumbed to infection by the 9th day post *P. berghei* infection. The majority of infected mice treated with PBS or IgG antibody succumbed to *P. berghei* infection between the 6th and 7th day post parasite inoculation (Fig. 13).

## Discussion

Limited understanding of human immunity to malaria infection constitute constraints that forestall efforts aimed at controlling disease progression and impede development of clinically efficacious malaria vaccine formulations [26]. *Plasmodium berghei* infection in rodents is widely employed albeit controversially as a means to better understand the pathomechanistic factors contributing to mortality from cerebral malaria [22]. Likewise, cytokine dysregulation has been recognized as the major cause of several immunopathological events that occur during malaria infection [27]. Herein, a model of *P. berghei* induced malaria infection was employed to investigate for the first time, the involvement of interleukin 35 (IL-35) in malaria infection.

The results obtained showed that *P. berghei* infection substantially elevated IL-35 expression in infected mice and the elevated concentration of IL-35 were observed to correlate significantly with parasitaemia progression. Elevation of serum IL-35 observed in this study corroborate earlier reports of systemic elevation of circulating levels of IL-35 observed in other infectious diseases including *Trichuris muris* infection and sepsis [16, 28]. A possible explanation for the observed elevation of IL-35 expression could be due to increased numbers of functional Foxp3^+^ regulatory T lymphocytes (Tregs) the primary cellular source of the novel anti-inflammatory (and immunosuppressive) cytokine IL-35 which are reportedly upregulated during malaria infection [7, 11, 29]. Regulatory T cells are recognized for their suppressive capacity which is crucial for the maintenance of self-tolerance and beneficial in the resolution of autoimmune disorders associated with chronic inflammation [7].

Malaria patients have been shown to exhibit enhanced Treg cell proportions in peripheral circulation as demonstrated by previous studies reporting increased numbers of Tregs in peripheral circulation of human subjects and their association with an increased risk of developing severe malaria [7, 30]. Regulatory T cells have been implicated albeit unfavourably in the suppression of beneficial anti-parasitic and anti-viral immune responses contributing to the failure of adequate infection control [11, 31]. A study involving regulatory T cells during malaria showed that infected mice were afforded protection from cerebral malaria and typically featured decreased parasite burdens coupled with lesser tendency towards parasite sequestration once Tregs had been depleted [7, 32]. Furthermore mice depleted of CD4^+^CD25^+^ Tregs were resistant to experimental cerebral malaria. This implies that neutralizing the suppressive actions of Tregs confers protection upon mice previously susceptible to ECM [32].

In addition a positive feedback mechanism associated with IL-35 signalling was reported in a study involving colorectal cancer patients [33]. The positive feedback loop promotes the generation of inducible regulatory T cell which mediate their suppressive actions exclusively via IL-35 (iTr-35) encouraging the generation of more IL-35 [33, 34]. This may possibly have contributed to the elevation of IL-35 expression that was observed in *P. berghei* infected mice.

The expression of IL-35p35 and possibly IL-35 was observed in all the organs assessed namely; the brain, liver, spleen, kidney, heart and lung. This may have been due to the highly inducible nature reported of the p35 and EBI3 subunits constituting IL-35 in response to inflammatory stimuli [34]. In addition to neurons of the cerebral cortex in brain sections from *P. berghei* infected mice (Fig. 3a), mild immunoreactivity for IL-35p35 was observed in sections of liver tissue from *P. berghei* infected mice and was localized to stellate macrophages (von Kupffer cells). Kupffer cells of hepatic sinusoids were observed to be generally enlarged and hyperplastic. Epithelial cells lining the portal tract of the liver also demonstrated positive staining for IL-35p35 (Fig. 3b).

Tissue expression of IL-35 was particularly dense in the spleen of *P. berghei* infected mice (Fig. 3c). The spleen represents a privileged site for the clearance of parasitized erythrocytes and priming of *Plasmodium* sp. specific T-lymphocytes in malaria [35]. The vital role the spleen plays during malaria infection has been associated with the generation of immune responses by resident T lymphocyte, B lymphocytes and macrophages, these cells being crucial cellular sources of IL-35 may have possibly accounted for the intense expression of IL-35 that was observed in the spleen of *P. berghei* infected mice. Furthermore, an increase in the population of CD4^+^CD25^high^ Foxp3^+^T cells (a known cellular sources of IL-35) in the spleen of *P. berghei* infected C57BL/6 mice has been documented [36].

The heart tissue demonstrated weak staining for IL-35p35 (Fig. 3f) a possible explanation may be due to the absence of the signal transducing receptor IL-12Rβ2 (an IL-35 receptor) in the heart according to a previous report [34]. However, in addition to IL-12Rβ2, IL-35 has been reported to also exert its action (albeit with partial loss of suppressive activity) via the ubiquitously expressed gp130 receptor [20]. The kidneys of *P. berghei* infected mice showed distinct immunolabeling for IL-35p35 (Fig. 3d). Staining was localized to the intercalated cells of the medullary papillary collecting ducts, epithelial cells lining the distal and proximal convoluted tubules while mild expression was observed in the macula densa region of the glomerulus. Marginal expression of IL-35p35 was observed in uninfected control mouse kidney and this was attributed to the constitutive expression profile of IL-35 in mouse tissue [34]. Studies characterizing the expression profile(s) of IL-35 in the kidney of malaria infected mice are limited. Nevertheless an experimental model of acute kidney injury during sepsis showed the capacity of renal tubular epithelial cells to secrete IL-35 [37].

Consistent with the patterns of IL-35p35 expression in the protein atlas [38], sections of lung tissue from *P. berghei* infected mice on day 1 following infection revealed initially weak cytoplasmic immunolabeling for IL-35p35 and possibly IL-35 in alveolar macrophages and alveolar epithelial cells which became characteristically more intense by the 5th day of *P. berghei* infection (Fig. 3d). There was no detectable expression of IL-35p35 in the lung tissues from uninfected mice.

Literature pertaining to the dynamics of IL-35 during malaria infection are limited, hence the effects of modulating IL-35 during *P. berghei* infection in mice was investigated. In addition to similar parasite burdens observed between infected mice treated with recombinant IL-35 protein and their PBS treated counterparts, a tendency towards mortality arising as early as the 4th day post *P. berghei* infection in recombinant IL-35 treated mice was also apparent. Cytokine responses observed in *P. berghei* infected mice that received recombinant IL-35 protein were quite similar to cytokine profiles demonstrated by their PBS treated counterparts (Fig. 12). Notably, levels of TNF, IFN-γ and IL-6 in recombinant IL-35 protein treated mice were comparable to those observed in PBS treated mice while expression of IL-10 was decreased and IL-2 increased in infected mice treated with recombinant IL-35 protein unlike their PBS treated counterparts. Infected mice treated with neutralizing AEBI3 antibody demonstrated markedly elevated levels of IFN-γ in addition to elevated levels of IL-10 and TNF compared to their IgG antibody treated counterparts (Fig. 12). However, the levels of IL-6 were significantly lower in *P. berghei* infected mice treated with neutralizing AEBI3 antibody when compared to their IgG antibody treated counterparts. Considering different concentration of neutralizing AEBI3 and IgG antibody were used it is probable this difference might have exerted some influence on the observed serum IL-6 cytokine levels. Previous studies have associated the simultaneous production of IFN-γ and IL-10 by effector T cells with the limitation of immunopathogenesis [39]. Importantly, protective responses from overt clinical malaria infection afforded by malaria vaccine formulations (circumsporozoite-based vaccines such as the RTS,S malaria vaccine candidate) are characterized by intense expression of IFN-γ [39].

Infected mice that received neutralizing AEBI3 antibody demonstrated generally lower parasitaemia peaks when compared to IgG antibody treated mice (Fig. 4). This observation could possibly be attributed to their enhanced secretion of IFN-γ (Fig. 12). IFN-γ produced by CD4^+^T cells reportedly exerts potent anti-parasitic actions in *Plasmodium* sp. infection [39]. These responses reportedly encompass the activation of cytotoxic CD8^+^ T cells, activation of PRBC phagocytosis by macrophages, nitric oxide mediated inhibition of parasite proliferation within hepatocytes, opsonisation of free merozoites and antibody-mediated parasite elimination [39]. IFN-γ receptor knockout mice or in vivo depletion of IFN-γ apparently featured high parasitaemia and increased tendency towards early mortality [40]. Hence it was surmised that the potentiation of IFN-γ release in *P. berghei* infected mice following the neutralization of IL-35 by treatment with neutralizing AEBI3 antibody afforded protection from high parasitaemia during *P. berghei* infection via the aforementioned actions of IFN-γ on parasite development.

The non-significant levels of IL-4 observed in this study could possibly be attributed to the fact that the study was based on primary infection with *P. berghei* while IL-4 (a Th2 cytokine) has been associated with malarial immunity to reinfection [40]. There is evidence that IL-4 and other Th2 cytokines play protective roles in re-infection than in primary infections [40], IL-4 knockout mice demonstrated optimal control of primary infection with *P. chabaudi chabaudi*, but had a more exacerbated disease upon reinfection [40]. Hence the observed lack of significant elevation in serum IL-4 levels across all the treatment groups was attributed to the study being centred around a primary infection as opposed to a secondary infection (or re-infection) where this cytokine (IL-4) reportedly partakes in the immune response(s).

## Conclusion

The study demonstrated that IL-35 expression in *P. berghei* infected mice was significantly elevated and correlated positively with increasing parasitaemia hinting at the involvement of IL-35 in the pathogenesis of malaria infection. The results of obtained indicate that the neutralization of IL-35 delayed parasitaemia progression in *P. berghei* infection, improved histological outcomes and promoted survival of *P. berghei* infected mice. These findings indicate that therapeutic strategies aimed at blocking the actions of IL-35 independently or as an adjuvant to existing anti-malarial therapies may offer benefit in ameliorating severe malaria infection.

## Data Availability

The datasets generated and analysed during the course of the current study are available from the corresponding author on reasonable request.

## Abbreviations

AEBI3: anti Epstein Barr induced gene 3
CTLA 4: cytotoxic T lymphocyte antigen 4
DAB: diaminobenzidine
ELISA: enzyme linked immunosorbent assay
GPI: glycosylphosphatidylinositol
HRP: horse radish peroxidase
IFN-γ: interferon gamma
IL: interleukin
NO: nitric oxide
PAMP: pathogen associated molecular pattern
PD 1: programmed cell death protein
PRBC: parasitized red blood cells
rIL-35: recombinant mouse IL-35 protein
SEM: standard error of the mean
STAT: signal transducer and activator of transcription
Th: helper T cell
TNF: tumour necrosis factor
Tregs: regulatory T cells
